# Analysis of Homogel Uniaxial Compression Strength on Bio Grouting Material

**DOI:** 10.3390/ma9040244

**Published:** 2016-03-29

**Authors:** Kyungho Park, Daehyeon Kim

**Affiliations:** Department of Civil Engineering, Chosun University, Gwangju 501-759, Korea; munhakng@gmail.com

**Keywords:** bio grouting, uniaxial compressive strength, calcium carbonate, microbial reaction

## Abstract

This study analyzed uniaxial compression strength over time by preparing a homogel specimen from a bio grouting material, a cement-like form produced by environment-friendly microbial reactions. Among chemical grouting methods, the most commonly used method is the Labile Waterglass method. In this study, the homogel uniaxial compressive strength of Labile Waterglass (LW) injection material and that of bio grouting material were measured and analyzed. In order to perform the experiment, a total of 10 types of grouting mixing ratios were prepared by a combination of different materials such as Ordinary Portland Cement, Micro Cement, Bio Grouting Material and Sodium Silicate. They were cured in the air, and their homogel uniaxial compression strengths were measured on days 1, 3, 7 and 28 Based on the test results, it was confirmed that the uniaxial strength of the specimen made with Bio Grouting Material, Ordinary Portland Cement and Micro Cement was increased by more than 30% than that of the specimen only used with Ordinary Portland Cement, as a result of hydrogen-released heat reaction between calcium carbonate, the main ingredient of the bio grouting material, and calcium silicate in the cement. This indicates that the use of 30% bio-grouting material instead of cement in the grouting can be a reasonable mixing ratio to save the use of cement, leading to reduction in CO_2_ emission.

## 1. Introduction

### 1.1. Background and Objective

It is difficult to secure grounds in Korea due to increasing costs of raw material led by the depletion of natural materials and the expansion of national key industry resulting from industrial development. Therefore, soft ground not previously utilized as construction sites increasingly attracts attention to efficiently use land. Soft ground, coastal dredged, and reclaimed grounds are used for construction sites as a base ground. Therefore, significant attention is now given to the method for improving soft ground that contains loose sandy soil or silt, not typically considered as a construction site before. 

Among other methods, in civil engineering projects, the grouting method has been used for reinforcing, repairing or waterproofing dams, slopes, embankments, dredged and reclaimed areas. Now, it is applied to almost every construction site, some examples being construction of expressways, airfields, express railways, undersea facilities, ports, tunnels, and subways. This implies the grouting method is now essential in construction sites. 

For the grouting method employed in construction sites of Korea, 1–3 types of liquid waterglass-based chemicals, urethane, or high-pressure jet grouting are employed. However, most of them focus on enhancing ground strength, and do not suggest specific solutions for environmental issues, including discharged CO_2_ and groundwater pollution due to cement and chemical liquid used as a raw material in grouting. Accordingly, a significant amount of funds is invested into developing new methods in the field of ground engineering in relation to the method of improving soft ground. There is a great need for a method that addresses the environmental issues in the process of improving soft ground.

The grouting method has had some issues caused by grout materials, and researchers have studied how to address the problems. The method generally employed in grouting with chemical liquid is the Labile Waterglass (LW) method which is effective for grouting into the gravel and sandy (larger than 0.6 mm) layers, but not effective for grouting into fine sand (*i.e.*, smaller than 0.6 mm) layers (Ahn [1]). The major material used in the LW method is cement, of which disadvantages include gelation and low levels of permeation. However, recent development of materials, cement crushing and classification technology includes new cement-based grouts, for example, micro cement, quick-hardening cement (Kim *et al.* [2]), and plastic cement (Kim *et al.* [3]). Their anticipated effect is improved cement gelation and permeation (Kim *et al.* [4]). Advantages of the LW method are to increase water resistance and adhesion between the soils and waterglass-based grouts. The fast flow of ground water can wash out the chemical liquids used in the grouting. Therefore, for the LW method, it is reported that raising the chemical liquid concentration, and the injection rate and speed, with shorter gel time, if it is applied to running water ground, enhances its efficiency.

The Korea Cement Association states that the volume of cement produced in Korea in 2006 is about 48 million tons, which is seventh place for cement production in the world. To produce one ton of cement, about 0.9 tons of CO_2_ are discharged. If a material that can replace 1% of 48 million tons of cement were developed, about 480 thousand tons of cement would be saved (Korea Cement Association [5]). This results in reducing the cost of approximately 13 billion won/year ($30 per ton of CO_2_ for the carbon credit). Korea is a participant in reducing CO_2_ emission in accordance with the Kyoto Protocol 2015. It is thus necessary to develop environment-friendly materials to replace cement or reduce the use of cement in the ground improvement field where cement is a major material to contribute to the green growth policy, for example, combating global warming. It is also necessary to address the issues of increasing raw material costs and lack of construction materials by developing new materials.

For this reason, in this study, Bio Grouting Material consisting of CaCO_3_ was produced by biochemical reaction of microorganism *Sporosarcina Pasteurii* (KCTC 3558) and Calcium Chloride, and was made with a form of powder-like cement. The following ten mixing ratios used for the conventional LW method and bio grouting method were prepared: OPC (Ordinary Portland Cement, hereinafter referred to as “OPC”) 100%; Micro (Micro Cement, hereinafter referred to as “Micro”) 100%; Bio (Bio Grouting Material, hereinafter to as “Bio”) 100%; OPC 100% + sodium silicate No. 3; Micro 100% + sodium silicate No. 3; Bio 100% + sodium silicate No. 3; OPC 100%+Bio 30%; OPC 70% + Bio 30%; Micro 100% + Bio 30%; and Micro 70% + Bio 30%. Then, the uniaxial compression strengths of these homogel mixtures were measured on days 1, 3, 7 and 28 of curing, and compared.

### 1.2. Previous Studies

Various studies about cementation of soil using microorganisms have been conducted over the last decade. Among numerous microorganisms present in the soil, many researchers chose *Sporosarcina Pasteurii* in particular to study cementation of soft grounds using the Bio grouting material produced through their biochemical reactions (Kim [6]; Park [7]; Kim *et al.* [8,9]; Park and Kim [10,11]; Jeon [12]; Mitchell and Santamarina [13]; Dejong *et al.* [14]).

The method, which enhances the strength of soil by mixing the Bio grouting material into loose sandy soil is named the Microbial Calcite Precipitation (MCP) method. The MCP method has been recognized as an environment-friendly method that can improve the strength of loose sandy grounds or improve soft grounds by having cement permeate the gaps between particles. In addition, a variety of studies for improving the grounds has been conducted using Biopolymer, organic materials, plant extracts and others, to fundamentally reduce the cement (Kim *et al.* [15]; Chang and Cho [16]).

It is confirmed that the MCP method improves the strengths of pure sandy grounds. However, a limitation exists in that the Bio grouting material needs to be injected several times in order to precipitate into loose or soft grounds and thus it is hard to apply in the field (Dejong *et al.* [14,17]; Soon *et al.* [18]).

Therefore, in order to ensure a more efficient and practical application of the MCP method, the eco-friendly microbial cementation method, the Bio grouting method, was proposed by combining the injection technology for soft ground treatment and the MCP method (Park and Kim [11]; Kim *et al.* [9]; Paassen *et al.* [19]; Wiffin *et al.* [20]; Whiffin [21]).

Park and Kim [11] confirmed the effects of cementation and grouting of loose sandy grounds with the Bio grouting material, produced through biochemical reactions of microbes. A grouting test was conducted by producing a two-solution, one-step grouting device by injecting a mixture of 2000 mL microbial solution and 2000 mL calcium chloride solution using the device into a specimen with the diameter (D) of 5 cm and the height (H) of 10 cm, under conditions similar to the field. The test result showed the cementation range of approximately 5.4 cm and the cementation strength of 150 kPa in the drainage condition, confirming the potential of Bio grouting.

The bio grouting test was conducted using the two-solution one-step grouting device for a small specimen with the size of (D) 5 cm × (H) 10 cm, which was only a basic study that confirmed the grouting potential of the Bio grouting material. Therefore, it is necessary to analyze the soil behaviors according to the mixing ratio of Bio grouting material under different ground conditions for medium-sized ground specimens, and analyze the schematic field applicability by conducting the LW method and the Bio grouting method, which are the representatives of chemical grouting methods.

Internationally, studies using microorganisms have been actively conducted in Europe, the USA and Japan, and currently “bio grouting” is commonly used as a term for the technique that combines the MCP method and the grouting method.

In addition to studies on bio grouting, several studies on cement sandy or soft grounds with the Bio precipitating into the gaps between sand particles, by injecting microorganisms to the surface of the sand particles where calcium ions were concentrated, were conducted (Scholl *et al.* [22]; Torkzaban *et al.* [23]).

Representative previous studies that apply the bio grouting method include the following.

Whiffin [22] made a cylindrical specimen with PVC with the volume of 6 L and the dimension of 66 m (diameter D) × 5 m (length L), and filled it with Itterbeck sand (D60/D10, the grain-diameter ratio corresponding to 60% and 10% of the soil grain size distribution curve) with the same relative density (DR). After 6 L of microbial solution was injected to the specimen, 6 L of calcium chloride solution (0.05 M) was injected in order to settle down the microorganisms evenly throughout the entire sand column. Then, 1 M of urea and 9 L of calcium chloride solution was injected repeatedly until urease reaction stopped. Afterwards, the sand column was left for 24 h so that the urease could react. Then, the concentration of the discharge solution was measured using a turbid meter. Both the microbial solution and the calcium chloride solution were injected freely over the length of 5 m a low pressure (approximately 7 m/day).

As a result of the experiment, a uniaxial compression strength value of 100 kg/cm^2^ was obtained using the strength transformation formula for the non-destructive testing ultrasonic velocity. However, as this is a uniaxial compression strength based on estimation, not obtained through quantitative analysis, it is not highly reliable. In addition, as a result of periodically injecting the microbial solution and the calcium chloride solution, the precipitation of calcium carbonate was created throughout the 5 m sand column, mostly around the inlet section and proportionally reduced depending on the distance from the inlet. This confirms that the calcium carbonate precipitation occurs when the microbial solution and the calcium chloride solution are mixed and the strength varies with the amount of calcium carbonate precipitation.

Paassen *et al.* [20] carried out an experiment to investigate the field applicability by filling a container box (0.9 m (width B) × 1.1 m (length L) × and 1 m (height H)) with the same relative density. Onto the inner sides of the container box, drainage screens were installed and at its bottom, a drainage device was installed. Using this container, the grouting experiments were conducted with Mass river sand (D60/D10 = 1.6) and IItterbeck sand (D60/D10 = 1.64).

Mass river sand was filled into the container box through free fall using a forklift at the same height in order to maintain the same relative density (*D*_R_) inside the container box. Then, 100 L of the microbial solution and the calcium chloride solution (0.5 M) were injected from the center to the sides of the box at a constant injection pressure of 50 L/h. Over a period of 50 days, 3500 L of the reaction solution was injected in eigth installments, and 200 M of the calcium carbonate (20 kg/m^3^) was precipitated at an efficiency of approximately 12%. The experiment confirmed that calcium carbonate was precipitated along with the bottom edges and the inner sides of the container.

According to Hamdan [24], chemical reactions of MICP involved in bacterial ureolysis produce undesirable and potentially toxic end products: ammonia (NH_3_(g)) and ammonium (NH_4_^+^). He stated that it is unclear whether or not a substantial portion of ammonium can be converted to nitrate in comparison to the amount produced, since nitrifying bacteria are limited by the lack of dissolved oxygen and may also experience severe inhibition at elevated ammonia concentrations, reported by Anthonisen *et al.* [25] and Antoniou *et al.* [26]. In addition, several studies have suggested that ammonium ions produced by the ureolysis reaction may exchange with radionuclide and metal contaminants sorbed to subsurface minerals, and this may enhance the availability of these contaminants for co-precipitation into calcite (Colwell [27]; Fujita *et al.* [28]; Fujita *et al.* [29]; Mitchell and Ferris [30]). Hamdan [24] pointed out that although the ion-exchange concept may be theoretically feasible, the studies have not done bench-scale or field-scale tests to verify the idea that ammonium, a water-soluble polyatomic ion, may sorb to minerals in exchange for radionuclide and metal contaminants. Although a number of studies on the MCP have been done, little attention has been given to treat ammonia (NH_3_(g)) and ammonium (NH_4_^+^), requiring further study regarding this topic. 

In order to develop such a bio grouting method, an engineering assessment of the grouting materials needs to be conducted first and, in this study, the engineering assessment of OPC, Micro, and Bio was conducted to apply the bio grouting to the field.

## 2. Preparation of Specimens and Types of Specimens

In this study, a method of using the Bio grouting material in place of chemical (sodium silicate No. 3), which could be a problem when the LW method was used, and the concept of admixture that could reduce the amount of cement by 30% were used. 

### 2.1. Mixing Ratio of the Bio Grouting Material for Uniaxial Compression Strength Test 

A mixture of grouting materials used for the conventional LW engineering method and the newly developed bio grouting material was made to measure the uniaxial compression strength of the developed grouting material. In order to evaluate its strength, specimens were prepared in homogenous gel type in cylindrical molds with the size of 5 cm (D) × 10 cm (H). After 3 h, they were taken out from the molds and cured by air drying and their strengths were measured on the first, third, seventh and 28th days after curing.

Mixing ratios of the grouting materials are as shown in Table 1. Solution A, an accelerator, and Solution B, a hardener, were mixed at 1:1 ratio through agitation and the mixture was cured at the prepared molds. Through the compression strength test, the homogenous gel cementation strength was measured according to ages of the grouting materials. The uniaxial compression strength was measured using a universal load tester, and the compression rate was measured at a rate of 1%/mm. Here, O refers to OPC; M, micro cement; B, bio grouting material; and S, sodium silicate No. 3. Of the numbers 1 and 2, 1 represents specimens in which the bio grouting material equivalent to 30% of cement was injected in place of sodium silicate No. 3, to compare the conventional LW method and the bio grouting method, and 2 represents the specimens made for uniaxial compression specimens in which the bio grouting material was injected as admixture with 30% reduced usage of cement.

### 2.2. Making of the Bio Grouting Material

**Figure 1:**
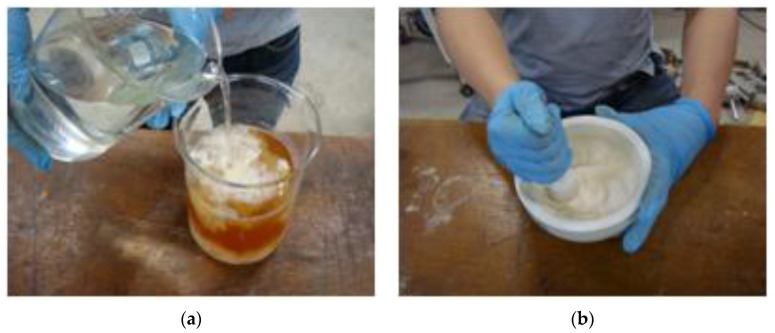
Making of the bio grouting material: (**a**) mix the microbial solution and the sodium silicate solution; and (**b**) make powder (calcium carbonate) in a bowl plate.

The bio grouting material used for this study was made by the same method as microbial solution used for typical concentration treatment as shown in Figure 1a. The microbial solution and the calcium chloride solution (0.75 M) were mixed at 1:1 ratio. When white precipitate was formed, only the sediment, which was bio grouting material, was extracted through a filter paper (Toyo filter paper with 18.5 in diameter and 0.6 μm in pore size). The extracted bio grouting material was dried for 24 h at 40 °C and made into powder (calcium carbonate) using a bowl plate, as shown in Figure 1b. The bio grouting material produced passed through a No. 200 sieve (75 μm). As the average particle size of CaCO_3_ (obtained from Microbial Induced Calcite Precipitation) ranges from 1–3 μm, the size of the grouting material used would be in the same range. Based on the XRD (X-Ray Diffraction) test, the extracted bio grouting material was found to be calcium carbonate. The microorganism used in the study, *Sporosarcina Pasteurii*, is most active at a pH level 9 and it does not proliferate (still alive for some time) at a pH level over 12. The microorganisms can survive although they are exposed to sodium silicate and Portland cement, as reported by the studies by Bundur *et al.* [31] and Jung [32]. In most studies, calcium carbonate is precipitated in the liquid, which is very difficult to use in the field. However, as the grout material developed in this study was produced like cement, it can be easily used in the field. This is what is unique about this study compared to other studies. 

**Figure 2:**
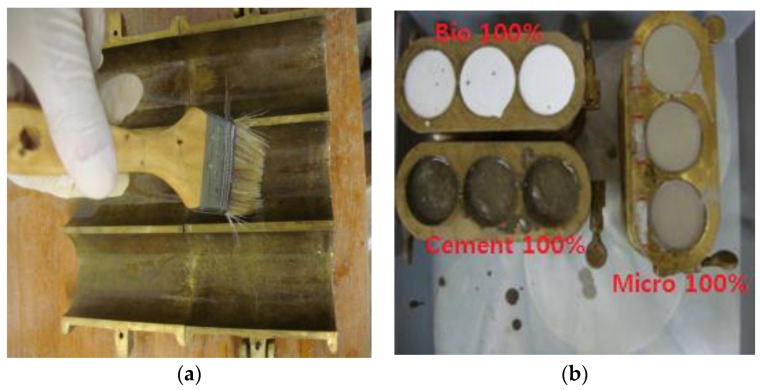
Making of homogenous gel specimens for the grouting material: (**a**) application of lubricant to specimen molds; and (**b**) curing.

Figure 2 shows the process of making specimens for grouting materials. Lubricant was applied to specimen molds that were separated into two halves, and then the injecting material was agitated according to the above mixing ratios. The agitated grouting material was put into the prepared molds. The specimens were taken out from the molds after six hours, and then cured through air drying. According to KS F 2403 [33], the average temperature and humidity in the lab were maintained with 20 ± 2 °C and 65% ± 20%, respectively. As the humidity in the study is lower than 100%, the strength obtained would be lower than that with 100% humidity after seven days.

**Figure 3:**
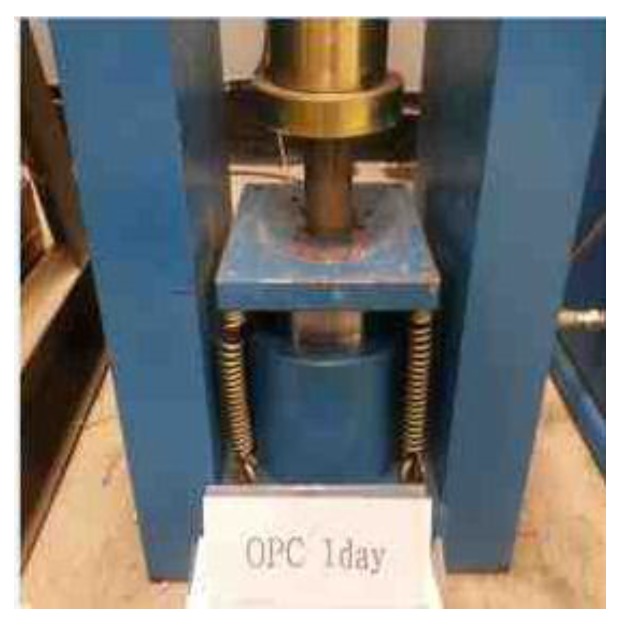
Test on formed OPC specimens.

The size of the specimens made was 5 cm (D) × 10 cm (H) and for analysis of the uniaxial compression strength, the specimens were cured for one, three, seven or 28 days. Then, the uniaxial compression test was conducted, as shown in Figure 3.

## 3. Result of Laboratory Tests

### 3.1. Result of the Uniaxial Compression Strength Test for the Grouting Material

#### 3.1.1. Evaluation of the uniaxial compression strengths of OPC, micro cement and bio grouting material

**Figure 4:**
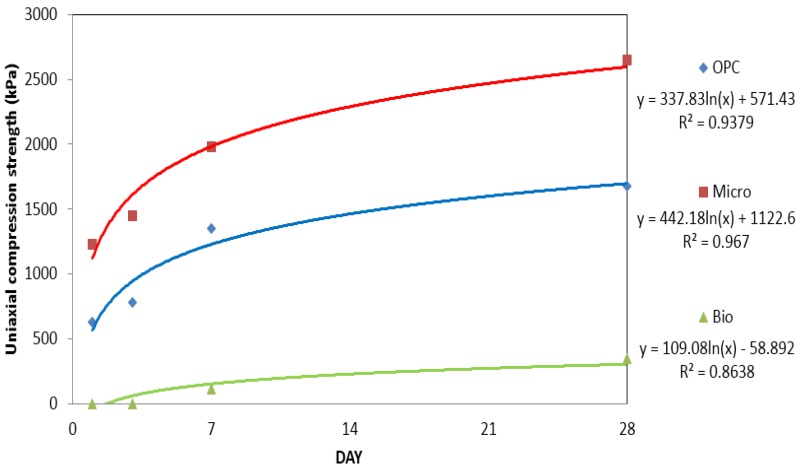
Uniaxial compression strength graphs for OPC, micro cement and bio grouting material.

Figure 4 and Table 2 compares the uniaxial compression strengths of OPC, micro cement and bio grouting materials over time. For OPC, the initial strength was measured as 630 kPa, which increased over time to 1350 kPa on the seventh day and 1680 kPa on the 28th day. For micro cement, the initial strength was measured as 1230 kPa, which increased over time to 1980 kPa on the seventh day and 2650 kPa on the 28th day, about double those of OPC. For OPC and micro cement, the increase of strength over time resulted from hydration of the cement. 

For the bio grouting material, strength could not be measured, as specimen was not yet formed. On the seventh day, its strength was measured as 110 kPa and on the 28th day, it was measured as 350 kPa. There was no hydration, but as indicated by the result of a study done by Jeon [12], strength was formed as water evaporated and the bio grouting material shrank. In order for bio grouting material to be useful, it should be used for the purpose of blocking water, as claimed by Kim and Park [34]. If it is to be used to improve strength, it should be used as admixture that could lead to further chemical reactions. 

#### 3.1.2. Uniaxial compression strengths of OS, MS and BS containing sodium silicate No. 3

**Figure 5:**
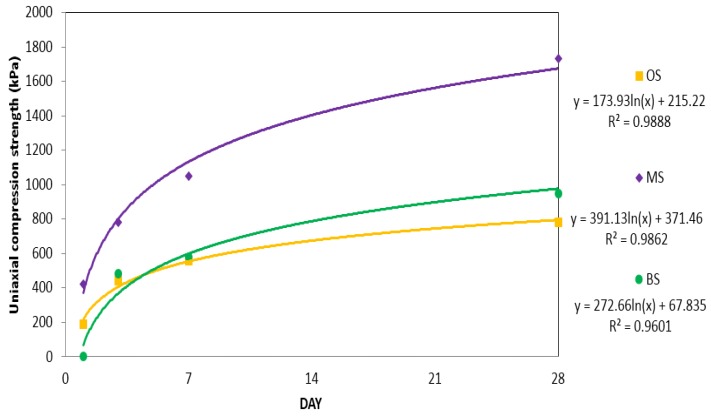
Uniaxial compression strength graphs for OS, MS and BS containing sodium silicate No. 3.

A comparison of the change of uniaxial compression strengths of OPC, micro cement and bio grouting material, into which sodium silicate No. 3 was mixed, over time, is shown in Figure 5 and Table 3. For OS, the initial strength was measured as 190 kPa, which increased over time to 560 kPa on the seventh day and 780 kPa on the 28th day. For MS, the initial strength was measured as 420 kPa, which increased over time to 1050 kPa on the seventh day and 1730 kPa on the 28th day, about double those of OS. 

For OS and MS, the increase of strength over time resulted from hydration of the cement. However, when compared to the strength of OPC and micro cement, the strength of specimens containing sodium silicate was found to be remarkably lower. As the sodium silicate No. 3 was a material used for the purpose of blocking water through quick cementation, rather than for the purpose of improving strength, cementation was very fast but hydration results in cracks in the specimen, making the strengthening effect insignificant. This result is similar to the result by Lee [35].

For the bio grouting material, strength could not be measured, as specimen was not yet formed. On the seventh day, its strength was measured as 580 kPa and on the 28th day, it was measured as 980 kPa, without any hydration. However, compared to the existing grouting material, it was confirmed that the bio grouting material formed adhesion because of sodium silicate No. 3. It was also confirmed that over time the bio grouting material shrank and the strength increased. 

#### 3.1.3. Uniaxial compression strengths of OPC and bio grouting material

**Figure 6:**
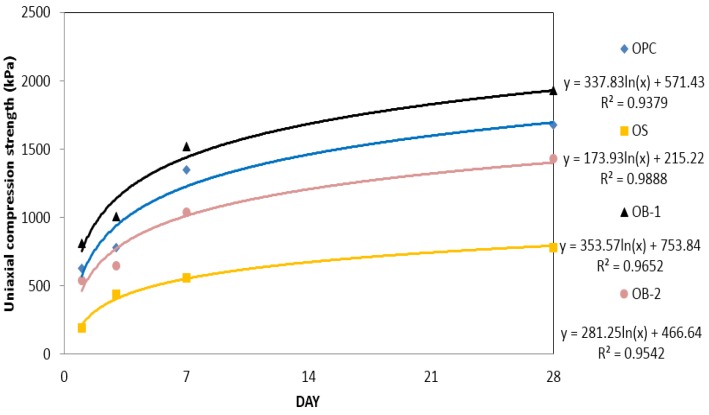
Uniaxial compression strength graphs for the improved OPC.

The measurement of uniaxial compression strength (see Figure 6) shows that OB-1 (OPC + bio grouting material) had the highest strength while that of OPC reduced by about 15% compared to OB-1. When OB-2 (OPC (70%) + bio grouting material (30%)) was used, the strength of OB-1 was measured to be about 15% lower than that of OPC. 

A comparison of the uniaxial compression strengths of OPC, OS, OB-1 and OB-2 is shown in Figure 6 and Table 4. As shown in Figure 6, for OPC, OS, OB-1 and OB-2, the compressive strength increased sharply up to approximately seven days, and then increased gradually. In order to evaluate the increase of the compressive strength with time depending on different mixing ratios, regression analyses were conducted using logarithmic function. The compressive strength of specimens with different mixing ratios can be estimated accurately using these regression equations, as the coefficients of regression are more than 0.93 for all the cases. 

#### 3.1.4. Uniaxial compression strengths of micro cement and bio grouting material

**Figure 7:**
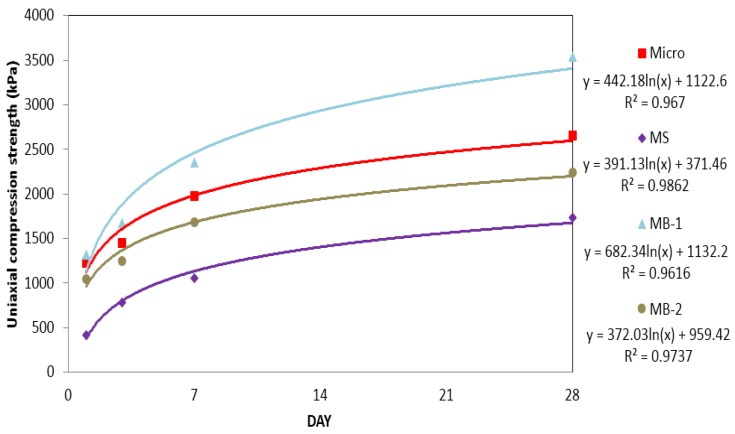
Uniaxial compression strength of the improved micro cement.

A comparison of the uniaxial compression strengths of micro cement, MS, MB-1, and MB-2 is shown in Figure 7 and Table 5. When measured for uniaxial compression strength, MB-1 (micro cement + bio grouting material) showed the highest strength, while micro cement had about 15% reduced strength compared to MB-1. When MB-2 (micro (70%) + bio grouting material (30%)) was used, the measured strength was found to be about 15% lower than that of micro cement. This result is similar to the result by Jeon [12], where the use of 30% bio grouting material produced a strength increase of about 15%.

Based on regression analyses for Micro, MS, MB-1 and MB-2, the regression coefficients are more than 0.97. This implies that the correlation between compressive strength and time is very high. 

When the bio grouting material was added to cement, there was a tendency that setting time was promoted and the initial strength increased. This may be due to promoted hydration by the reaction between calcium carbonate (main ingredient of the bio grouting material) and calcium silicate in cement. This observation was similar to the results by Kim and Noh [36] and Daimon [37]. 

## 4. Conclusions

In this study, an environment-friendly bio grouting material was prepared in powder form using the MCP method. Untreated and treated specimens, O, M, B, OS, MS, BS, OB-1, OB-2, MB-1 and MB-2, were cured for the periods of one, three, seven and 28 days age. Through the uniaxial compression tests for those specimens, the following conclusions were made.

1. Measurements of the homogel uniaxial compression strengths show that OPC 100% + Bio 30% and Micro 100% + Bio have the greatest strength. It is confirmed that the strength of both OPC 100% and Micro 100% is about 15% lower than that of the aforementioned two specimens. It is also confirmed that the strength of OPC 70% + Bio 30% and Micro 70% + Bio 30% is reduced by 15% compared with that of the latter two specimens. This indicates that the use of 30% bio-grouting material instead of cement in the grouting can be a reasonable mixing ratio to save the use of cement, leading to reduction in CO_2_ emission.
2. Calcium carbonate, the main component of the bio grouting material, reacts with calcium silicate in cement to release hydration heat, leading to increased strength. In order to use bio grouting, it has to be used for the purpose of blocking water. For its strength to be increased, the bio grouting material should be used as admixture.
3. In future studies, the strength, permeation range and gel time will be determined for different mixing ratios of Bio grouting material, OPC, Micro cement W/C (water/cement) ratio, and sodium silicate No. 3. It is expected that various mixing ratios of the bio grouting material created through microbial reactions can be determined depending on the conditions of the ground to be grouted.

## Figures and Tables

**Table 1:** Mixing ratios for uniaxial compressive strength test specimens for grouting materials.

Classification	Solution A			Solution B			
	Sodium Silicate No. 3 (mL)	Bio Grouting Material (g)	Water (mL)	OPC (g)	Micro Cement (g)	Bio Grouting Material (g)	Water (mL)
O	-	-	-	200	-	-	200
M	-	-	-	-	200	-	200
B	-	-	-	-	-	200	200
OS	65	-	-	200	-	-	200
MS	65	-	-	-	200	-	200
BS	65	-	-	-	-	200	200
OB-1	-	65	-	200	-	-	200
OB-2	-	65	-	135	-	-	200
MB-1	-	65	-	-	200	-	200
MB-2	-	65	-	-	135	-	200

Notes: O = OPC 100%, M = Micro 100%, B = Bio 100%; OS = OPC 100% + Sodium silicate No. 3; MS = Micro 100% + Sodium silicate No. 3; BS = Bio 100% + Sodium silicate No. 3; OB-1 = OPC 100% + Bio 30%; OB-2 = OPC 70% + Bio 30%; MB-1 = Micro 100% + Bio 30%; MB-2 = Micro 70% + Bio 30%.

**Table 2:** Uniaxial compression strengths of OPC, micro cement and bio grouting material.

Curing Time (day)	Uniaxial Compression Strength (kPa)		
	OPC	Micro Cement	Bio Grouting Material
1	630	1230	-
3	780	1450	100
7	1350	1980	110
28	1680	2650	300

**Table 3:** Uniaxial compression strengths of OS, MS and BS containing sodium silicate No. 3.

Curing Time (day)	Uniaxial Compression Strength (kPa)		
	OS	MS	BS
1	190	420	-
3	440	780	480
7	560	1050	580
28	780	1730	950

**Table 4:** Uniaxial compression strength of the improved OPC.

Curing Time (day)	Uniaxial Compression Strength (kPa)			
	OPC	OS	OB-1	OB-2
1	630	190	810	540
3	780	440	1010	650
7	1350	560	1520	1040
28	1680	780	1930	1430

**Table 5:** Uniaxial compression strengths of the improved micro cement.

Curing Time (day)	Uniaxial Compression Strength (kPa)			
	Micro	MS	MB-1	MB-2
1	1230	420	1320	1040
3	1450	780	1670	1250
7	1980	1050	2350	1680
28	2650	1730	3540	2240
